# Editorial: Editors' showcase: molecular signalling and pathways

**DOI:** 10.3389/fnmol.2024.1479278

**Published:** 2024-08-29

**Authors:** Jean-Marc Taymans, Deniz Yilmazer-Hanke

**Affiliations:** ^1^University of Lille, Inserm, CHU Lille, UMR-S 1172 - LilNCog - Lille Neuroscience & Cognition, Lille, France; ^2^Clinical Neuroanatomy, Neurology, University Hospital Ulm, University of Ulm, Ulm, Germany

**Keywords:** complexin, human iPSC-derived neurons, TRP channelopathies, miRNA—microRNA, STING, sterol regulatory element-binding protein (SREBP) pathway, subarachnoid hemorrhage, neurodegenerative diseases

Understanding molecular signaling and pathways is key for understanding brain function in health and disease. In this editor's showcase, we present selected research articles recently published in Frontiers in Molecular Neuroscience Section Molecular Signalling and Pathways from emerging and interesting fields with an emphasis on molecular neuroscience. Two articles entail original research on model systems, one on neuronal networks derived from human induced pluripotent stem cells (iPSCs) grown on high density arrays, in which the balance of excitatory or inhibitory neural mechanisms was studied, and one on the retina, developmentally a derivative of the human brain, to exemplify players in presynaptic adaptation of transmitter release in the brain. Three review articles dwell into molecular mechanisms with high therapeutic potential in human diseases, including transient receptor potential (TRP) channels, exosomal miRNAs, innate immunity, and lipid biosynthesis. These mechanisms are discussed in the context of highly relevant human diseases such as acquired and hereditary channelopathies, subarachnoid hemorrhage, and neurodegeneration.

Parodi et al. created a novel *in vitro* model of the human brain using high-density Micro-Electrode Arrays (MEA), where they examined the “excitatory/inhibitory balance” in human-iPSCs-derived neuronal networks. For modeling pathological changes seen in neurodegenerative and neurodevelopmental diseases, the authors generated arrays with 2304 electrodes with different neuronal compositions of excitatory glutamatergic and inhibitory GABAergic neurons, studying two neuronal culture configurations: 100% glutamatergic (100E) and 75% glutamatergic/25% GABAergic (75E/25I) neurons. At 56 days *in vitro*, when the GABA shift had occurred, they first characterized spontaneous electrophysiological activity. Electrical stimulation showed that 100E responded reliably, while 75E/25I required more tuning. Chemical stimulation with the GABAA receptor antagonist bicuculine increased firing and bursting only in 75E/25I, whereas the NMDA receptor antagonist APV and the AMPA/kainate receptor antagonist CNQX significantly altered both configurations, emphasizing the importance of neuronal diversity in neural network dynamics in a human-based *in vitro* system.

Lux et al. investigated the adaptation of the synaptic sensitivity of retinal rod photoreceptors to varying light intensities by focusing on complexins, which are SNARE complex regulators. Complexin 4 (Cplx4) was identified as the predominant complexin in the light-dependent regulation of neurotransmitter release. In ribbon synapses of rod photoreceptors, fewer synaptic vesicles were available for release in light than in dark in wildtype mice compared to Cplx4-deficient mice. Electrophysiology revealed that Cplx4 reduces or clamps Ca^2+^-dependent sustained release of synaptic vesicles, enhancing light signaling, likely by preventing desensitization. A proteomic screen identified Transducin as a Cplx4-SNARE complex interactor, suggesting a presynaptic interplay that facilitates light adaptation in rod photoreceptors, offering novel insights into photoreceptor function in varying lighting conditions.

Huang et al.'s article, resulting from academia-industrial collaboration, reviews the therapeutic potential of TRP channelopathies in drug discovery and clinical trials, along with their structure and function. After addressing the role of TRP channels in hereditary channelopathies, the focus shifts to acquired TRP-related diseases. They first discuss the TRP ion channel superfamily in humans, consisting of 27 members and divided into six subfamilies—TRPC (canonical), TRPV (vanilloid), TRPM (melastatin), TRPA (ankyrin), TRPML (mucolipin), and TRPP (polycystins). These channels act as multifunctional signaling proteins, triggering physiological responses to various stimuli. For example, TRPV1 is involved in thermoregulation, and TRPM8 in cold sensing. Specific TRP channels are also activated by natural products like capsaicin, cannabinoids, menthol, and wasabi compounds. Authors detail mutations in TRP channels linked to different channelopathies, including TRPV4-related peripheral neuropathies and skeletal disorders. Among acquired TRP channelopathies, they explore analgesic effects of agonists and antagonists of the TRP channels TRPV1-4, TRPA1, and TRPM8 involved in pain and richly expressed in sensory neurons. In respiratory diseases, they present inhibitors of the channels TRPV1, TRPA1, and TRPV4 expressed in lung cells, which are promising in models of asthma and chronic obstructive pulmonary disease (COPD). Broader implications of TRP channels in other acquired diseases, including ischemia/reperfusion injuries, anxiety, itching, cardiovascular diseases, diabetes, and cancers, are also discussed.

Liao et al. review the impact of exosomal miRNAs in subarachnoid hemorrhage (SAH), including neuronal apoptosis, immune activation, and inflammatory responses, and they highlight potential clinical applications of exosomal miRNAs in SAH treatment. Authors first describe exosomes as a type of extracellular vesicles derived from multivesicular bodies (MVBs) with < 120 nm diameter, and marked by CD63, MHC class II, and heat shock proteins. They outline paracrine and endocrine functions of exosomes in cellular processes, after being secreted by various cells (immune, brain, fat, tumor) to body fluids, including blood and cerebrospinal fluid. After detailing exosome's biogenesis, contents (nucleic acids, lipids, metabolic products, and proteins), and isolation for research purposes, they focus on exosomal miRNAs that are non-coding RNAs. They highlight miRNA's cellular synthesis and release, and their role in the regulation of gene expression, affecting cell differentiation, immune responses, and tumorigenesis, and diseases like chronic hepatitis, atherosclerosis, diabetes. Then, the article summarizes specifically recent research progress on the field of exosomal miRNAs in SAH.

The authors Scoles and Pulst delve into the complex interplay between the STING pathway, involved in immune responses activated by nucleic acids, and the cholesterol and fatty acid synthesis pathway regulated by SREBP, with a focus on neurodegenerative diseases. They further highlight the protein INSIG1 that anchors STING, SREBP and SCAP at the endoplasmic reticulum, when sterols are abundant. This allows INSIG1 to act as a regulatory hub that balances the two pathways: when STING-mediated innate immunity is activated, SREBP-mediated cholesterol and fatty acid synthesis are suppressed, and vice versa. This regulation becomes critical in neurodegenerative diseases, where chronic STING activation due to cellular damage disrupts lipid metabolism, leading to neuroinflammation, autophagy failure, and disease progression. The role of STING as a proton channel, which, when activated, can lead to cell death, is also discussed. The inverse relationship of STING and SREBP contributing to abnormal signaling in neurodegenerative diseases is considered for new therapeutic strategies.

By bringing together original data from diverse research fields and reviews with different perspectives, we hope to capture and stimulate the interest of our colleagues for research at the forefront of molecular signaling and pathways.

